# *N*-[3-(Benzyl­dimethyl­aza­nium­yl)prop­yl]-*N*′,*N*′,*N*′′,*N*′′-tetra­methyl­guanidinium bis­(tetra­phenyl­borate)

**DOI:** 10.1107/S1600536813012786

**Published:** 2013-05-18

**Authors:** Ioannis Tiritiris

**Affiliations:** a Fakultät Chemie/Organische Chemie, Hochschule Aalen, Beethovenstrasse 1, D-73430 Aalen, Germany

## Abstract

In the crystal structure of the title salt, C_17_H_32_N_4_^2+^·2C_24_H_20_B^−^, the C—N bond lengths in the CN_3_ unit of the guanidinium ion are 1.323 (4), 1.336 (5) and 1.337 (5) Å, indicating partial double-bond character in each. The C atom of this unit is bonded to the three N atoms in a nearly ideal trigonal–planar geometry [N—C—N angles = 117.7 (4), 120.9 (3) and 121.4 (3)°] and the positive charge is delocalized in the CN_3_ plane. The bonds between the N atoms and the terminal C-methyl groups of the guanidinium moiety all have values close to a typical single bond [1.452 (5)–1.484 (6) Å]. In the crystal, C—H⋯π inter­actions are present between guanidinium H atoms and the phenyl rings of both tetra­phenyl­borate ions. This leads to the formation of a two-dimensional supramolecular pattern along the *ab* plane.

## Related literature

For biosorption of tetra­decyl benzyl dimethyl ammonium chloride onto activated sludge, see: Ren *et al.* (2011 ▶). For the synthesis of *N*′′-[3-(di­methyl­amino)­prop­yl]-*N*,*N*,*N*′,*N′-*tetra­methyl­guanidinium chloride, see: Tiritiris & Kantlehner (2012 ▶). For the structures of alkali metal tetra­phenyl­borates, see: Behrens *et al.* (2012 ▶). For the structures of *N*,*N*,*N*′,*N*′,*N*′′-penta­methyl-*N*′′-[3-(tri­methyl­aza­nium­yl)prop­yl]guanidin­ium bis­(tetra­phenyl­borate) and *N*,*N*,*N*′,*N*′,*N*′′-tetra­methyl-*N*′′-[3-(tri­methyl­aza­nium­yl)prop­yl]guanidinium bis­(tetra­phenyl­borate) acetone disolvate, see: Tiritiris (2013*a* ▶,*b* ▶).
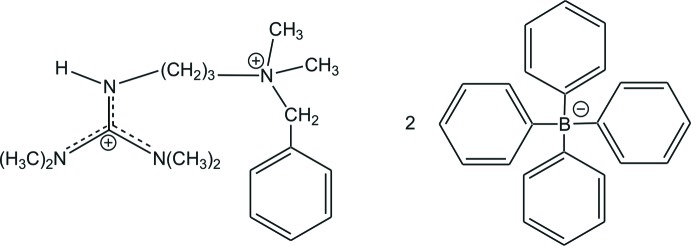


## Experimental

### 

#### Crystal data

C_17_H_32_N_4_^2+^·2C_24_H_20_B^−^
*M* *_r_* = 930.89
Monoclinic, 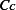

*a* = 17.1981 (3) Å
*b* = 17.3466 (3) Å
*c* = 17.8082 (4) Å
β = 94.182 (1)°
*V* = 5298.55 (18) Å^3^
*Z* = 4
Mo *K*α radiation
μ = 0.07 mm^−1^
*T* = 293 K
0.19 × 0.17 × 0.13 mm

#### Data collection

Bruker–Nonius KappaCCD diffractometer
6442 measured reflections
6442 independent reflections
5242 reflections with *I* > 2σ(*I*)

#### Refinement

*R*[*F* ^2^ > 2σ(*F* ^2^)] = 0.043
*wR*(*F* ^2^) = 0.110
*S* = 1.05
6442 reflections
650 parameters
2 restraints
H atoms treated by a mixture of independent and constrained refinement
Δρ_max_ = 0.17 e Å^−3^
Δρ_min_ = −0.15 e Å^−3^

### 

Data collection: *COLLECT* (Hooft, 2004 ▶); cell refinement: *SCALEPACK* (Otwinowski & Minor, 1997 ▶); data reduction: *SCALEPACK*; program(s) used to solve structure: *SHELXS97* (Sheldrick, 2008 ▶); program(s) used to refine structure: *SHELXL97* (Sheldrick, 2008 ▶); molecular graphics: *DIAMOND* (Brandenburg & Putz, 2005 ▶); software used to prepare material for publication: *SHELXL97*.

## Supplementary Material

Crystal structure: contains datablock(s) I, global. DOI: 10.1107/S1600536813012786/zl2550sup1.cif

Structure factors: contains datablock(s) I. DOI: 10.1107/S1600536813012786/zl2550Isup2.hkl

Additional supplementary materials:  crystallographic information; 3D view; checkCIF report

## Figures and Tables

**Table 1:** Hydrogen-bond geometry (Å, °) *Cg*1–*Cg*5 are the centroids of the C36–C41, C30–C35, C24–C29, C42–C47 and C60-C65 rings, respectively.

*D*—H⋯*A*	*D*—H	H⋯*A*	*D*⋯*A*	*D*—H⋯*A*
C11—H11*B*⋯*Cg*1	0.97	2.73	3.699 (2)	174
C11—H11*A*⋯*Cg*2	0.97	2.66	3.509 (2)	145
C14—H14⋯*Cg*3^i^	0.93	2.92	3.531 (2)	124
C9—H9*A*⋯*Cg*4^ii^	0.97	2.91	3.569 (2)	126
C7—H7*A*⋯*Cg*5^iii^	0.96	2.82	3.696 (2)	133

Symmetry codes: (i) 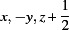; (ii) 
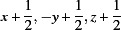
; (iii) 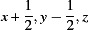.

**Fig. 1.**
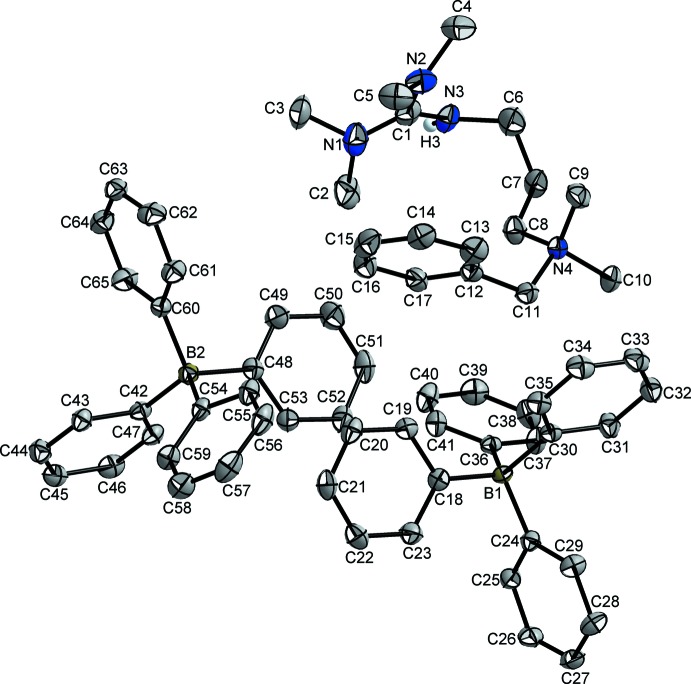
The structure of the title compound with displacement ellipsoids at the 30% probability level. All carbon bonded hydrogen atoms were omitted for the sake of clarity.

**Fig. 2.**
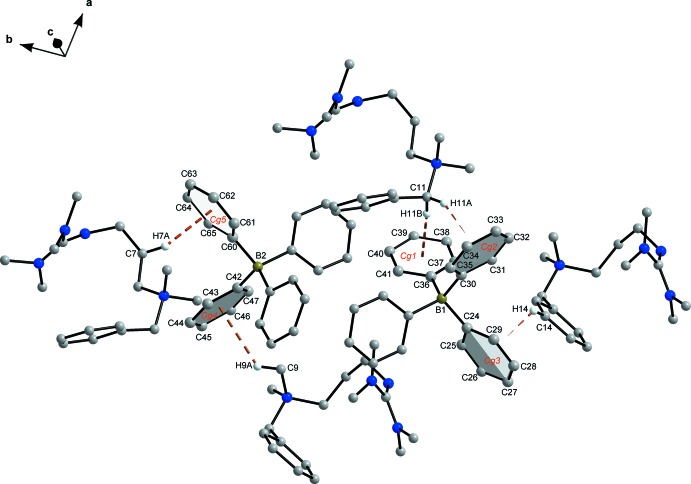
C–H···π interactions (brown dashed lines) between the hydrogen atoms of the guanidinium ion and the phenyl rings (centroids) of the tetraphenylborate ions.

## supplementary crystallographic information

### Comment

Alkyldimethylbenzylammonium salts with various even-numbered alkyl chain lengths are cationic surface-acting agents, belonging to the group of quaternary ammonium salts. They are used as biocides and also as phase transfer agents (Ren *et al.*, 2011), with the biocidal effect being due to damaging of the bacterial cell membrane and subsequent leakage of intracellular components. Based on our previous studies about dicationic ammonioalkyl guanidinium salts (Tiritiris, 2013*a* and 2013*b*), we synthesized the here presented title compound to investigate its biocidal properties. According to the structure analysis, the C1–N1 bond of the the CN_3_ unit is 1.336 (5) Å, C1–N2 = 1.323 (4) Å and C1–N3 = 1.337 (5) Å, showing partial double-bond character. The N–C1–N angles are: 121.4 (3)° (N1–C1–N2), 117.7 (4)° (N1–C1–N3) and 120.9 (3)° (N2–C1–N3), which indicates a nearly ideal trigonal-planar surrounding of the carbon centre by the nitrogen atoms. The positive charge is completely delocalized on the CN_3_ plane (Fig. 1). The bonds between the N atoms and the terminal C-methyl groups of the guanidinium moiety all have values close to a typical single bond [1.452 (5)–1.484 (6) Å]. The C–N bond lengths in the terminal benzyldimethylammonium group are slightly elongated [1.483 (4)–1.536 (3) Å]. The bond lengths and angles in the tetraphenylborate ions are in good agreement with the data from the crystal structure analysis of the alkali metal tetraphenylborates (Behrens *et al.*, 2012). Similar to the compound *N*,*N*,*N*',*N*',*N*''-tetramethyl-*N*''-[3-(trimethylazaniumyl)propyl]guanidinium bis(tetraphenylborate) acetone disolvate (Tiritiris, 2013*b*), C–H···π interactions between hydrogen atoms of the guanidinium ion and phenyl rings (centroids) of both tetraphenylborate ions are present, with bond lengths ranging from 2.66 to 2.92 Å (Table 1). Here, hydrogen atoms of –CH_2_ groups, –N(CH_3_) and C_Phenyl_ are involved (Fig. 2). In contrast, N–H···Ph interactions towards the (BPh_4_)^-^ ions were not observed.

### Experimental

The title compound was obtained by reaction of *N*''-[3-(dimethylamino)propyl]-*N*,*N*,*N*',*N*'-tetramethylguanidinium chloride (Tiritiris & Kantlehner, 2012) with one equivalent benzyl chloride in acetonitrile at room temperature. After evaporation of the solvent the crude *N*,*N*,*N*',*N*'-tetramethyl-*N*''-[3-(benzyldimethylammonio)propyl]guanidinium dichloride (I) was washed with diethylether and dried *in vacuo*. 1.0 g (2.75 mmol) of (I) was dissolved in 20 ml acetonitrile and 1.88 g (5.5 mmol) of sodium tetraphenylborate in 20 ml acetonitrile was added. After stirring for one hour at room temperature, the precipitated sodium chloride was filtered off. The title compound crystallized from a saturated acetonitrile solution after several days at 273 K, forming colorless single crystals. Yield: 1.97 g (77%). ^1^H NMR (500 MHz, CD_3_CN/TMS): δ = 1.90–2.03 (m, 2 H, –CH_2_), 2.78 [s, 6 H, –N(CH_3_)_2_], 2.85 [s, 12 H, –N(CH_3_)_2_], 3.01–3.08 (m, 4 H, –CH_2_), 4.26 (s, 2 H, –CH_2_), 5.95 (s, 1 H, –NH), 6.81–6.87 (t, 8H, –C_6_H_5_), 6.96–7.02 (t, 16 H, –C_6_H_5_), 7.24–7.31 (m, 16 H, –C_6_H_5_), 7.43–7.50 (m, 5 H, –C_6_H_5_). ^13^C NMR (125 MHz, CD_3_CN/TMS): δ = 22.6 (–CH_2_), 39.2 [–N(CH_3_)_2_], 42.8 (–CH_2_), 50.6 [–N(CH_3_)_2_], 62.2 (–CH_2_), 69.4 (–CH_2_), 122.8 (–C_6_H_5_), 126.6–126.7 (–C_6_H_5_), 129.0 (–C_6_H_5_), 131.9 (–C_6_H_5_), 132.7 (–C_6_H_5_), 135.4 (–C_6_H_5_), 161.1 (N_3_C^+^), 162.3–164.7 (–C_6_H_5_).

### Refinement

The title compound crystallizes in the non-centrosymmetric space group *Cc*; however, in the absence of significant anomalous scattering effects, the Flack parameter is essentially meaningless. Accordingly, Friedel pairs were merged. The N-bound H atom was located in a difference Fourier map and was refined freely [N—H = 0.85 (5) Å]. The hydrogen atoms of the methyl groups were allowed to rotate with a fixed angle around the C–N bond to best fit the experimental electron density, with *U*_iso_(H) set to 1.5 *U*_eq_(C) and d(C—H) = 0.96 Å. The remaining H atoms were placed in calculated positions with d(C—H) = 0.97 Å (H atoms in CH_2_ groups) and (C—H) = 0.93 Å (H atoms in aromatic rings). They were included in the refinement in the riding model approximation, with *U*_iso_(H) set to 1.2 *U*_eq_(C).

### Crystal data

**Table:**

C_17_H_32_N_4_^2+^·2C_24_H_20_B^−^	*F*(000) = 2000
*M**_r_* = 930.89	*D*_x_ = 1.167 Mg m^−^^3^
Monoclinic, *C**c*	Mo *K*α radiation, λ = 0.71073 Å
Hall symbol: C -2yc	Cell parameters from 6274 reflections
*a* = 17.1981 (3) Å	θ = 0.4–28.3°
*b* = 17.3466 (3) Å	µ = 0.07 mm^−^^1^
*c* = 17.8082 (4) Å	*T* = 293 K
β = 94.182 (1)°	Block, colorless
*V* = 5298.55 (18) Å^3^	0.19 × 0.17 × 0.13 mm
*Z* = 4	

### Data collection

**Table:**

Bruker–Nonius KappaCCD diffractometer	5242 reflections with *I* > 2σ(*I*)
Radiation source: sealed tube	*R*_int_ = 0.000
Graphite monochromator	θ_max_ = 28.2°, θ_min_ = 1.7°
φ scans, and ω scans	*h* = −22→22
6442 measured reflections	*k* = 0→23
6442 independent reflections	*l* = 0→23

### Refinement

**Table:**

Refinement on *F*^2^	Primary atom site location: structure-invariant direct methods
Least-squares matrix: full	Secondary atom site location: difference Fourier map
*R*[*F*^2^ > 2σ(*F*^2^)] = 0.043	Hydrogen site location: difference Fourier map
*wR*(*F*^2^) = 0.110	H atoms treated by a mixture of independent and constrained refinement
*S* = 1.05	*w* = 1/[σ^2^(*F*_o_^2^) + (0.0457*P*)^2^ + 1.9323*P*] where *P* = (*F*_o_^2^ + 2*F*_c_^2^)/3
6442 reflections	(Δ/σ)_max_ < 0.001
650 parameters	Δρ_max_ = 0.17 e Å^−^^3^
2 restraints	Δρ_min_ = −0.15 e Å^−^^3^

### Special details

**Table:**

Geometry. All e.s.d.'s (except the e.s.d. in the dihedral angle between two l.s. planes) are estimated using the full covariance matrix. The cell e.s.d.'s are taken into account individually in the estimation of e.s.d.'s in distances, angles and torsion angles; correlations between e.s.d.'s in cell parameters are only used when they are defined by crystal symmetry. An approximate (isotropic) treatment of cell e.s.d.'s is used for estimating e.s.d.'s involving l.s. planes.
Refinement. Refinement of *F*^2^ against ALL reflections. The weighted *R*-factor *wR* and goodness of fit *S* are based on *F*^2^, conventional *R*-factors *R* are based on *F*, with *F* set to zero for negative *F*^2^. The threshold expression of *F*^2^ > σ(*F*^2^) is used only for calculating *R*-factors(gt) *etc*. and is not relevant to the choice of reflections for refinement. *R*-factors based on *F*^2^ are statistically about twice as large as those based on *F*, and *R*- factors based on ALL data will be even larger.

### Fractional atomic coordinates and isotropic or equivalent isotropic displacement parameters (Å^2^)

**Table:**

	*x*	*y*	*z*	*U*_iso_*/*U*_eq_	
N1	0.5085 (2)	0.39414 (17)	0.5440 (2)	0.0773 (8)	
N2	0.57313 (17)	0.32797 (18)	0.64307 (18)	0.0702 (8)	
N3	0.61752 (19)	0.32381 (18)	0.5236 (2)	0.0755 (9)	
H3	0.622 (3)	0.355 (3)	0.487 (3)	0.097 (15)*	
N4	0.52643 (12)	0.09471 (13)	0.49726 (12)	0.0428 (5)	
C1	0.56661 (19)	0.34872 (17)	0.5714 (2)	0.0608 (8)	
C2	0.4793 (4)	0.3934 (3)	0.4647 (3)	0.1128 (17)	
H2A	0.4929	0.3454	0.4422	0.169*	
H2B	0.5022	0.4352	0.4388	0.169*	
H2C	0.4237	0.3990	0.4612	0.169*	
C3	0.4783 (4)	0.4576 (3)	0.5896 (4)	0.1197 (19)	
H3A	0.4252	0.4470	0.5994	0.180*	
H3B	0.4807	0.5052	0.5624	0.180*	
H3C	0.5094	0.4616	0.6364	0.180*	
C4	0.6487 (3)	0.3162 (3)	0.6859 (3)	0.0993 (15)	
H4A	0.6901	0.3293	0.6549	0.149*	
H4B	0.6536	0.2631	0.7008	0.149*	
H4C	0.6517	0.3484	0.7298	0.149*	
C5	0.5049 (3)	0.3181 (3)	0.6870 (3)	0.1009 (15)	
H5A	0.4586	0.3159	0.6536	0.151*	
H5B	0.5014	0.3608	0.7209	0.151*	
H5C	0.5100	0.2711	0.7154	0.151*	
C6	0.6576 (2)	0.2501 (2)	0.5253 (3)	0.0816 (11)	
H6A	0.6515	0.2253	0.5733	0.098*	
H6B	0.7129	0.2588	0.5211	0.098*	
C7	0.6271 (2)	0.1969 (2)	0.4623 (2)	0.0769 (11)	
H7A	0.6588	0.1506	0.4637	0.092*	
H7B	0.6329	0.2222	0.4145	0.092*	
C8	0.54227 (17)	0.17373 (18)	0.46620 (18)	0.0556 (7)	
H8A	0.5175	0.2115	0.4968	0.067*	
H8B	0.5170	0.1769	0.4158	0.067*	
C9	0.56539 (19)	0.0830 (2)	0.57348 (17)	0.0601 (8)	
H9A	0.5510	0.0336	0.5924	0.090*	
H9B	0.5494	0.1228	0.6066	0.090*	
H9C	0.6209	0.0851	0.5708	0.090*	
C10	0.55365 (19)	0.0334 (2)	0.44516 (19)	0.0628 (8)	
H10A	0.6092	0.0369	0.4432	0.094*	
H10B	0.5289	0.0409	0.3956	0.094*	
H10C	0.5402	−0.0165	0.4635	0.094*	
C11	0.43788 (15)	0.08420 (17)	0.49881 (17)	0.0509 (6)	
H11B	0.4147	0.0882	0.4477	0.061*	
H11A	0.4279	0.0325	0.5165	0.061*	
C12	0.39766 (14)	0.14053 (17)	0.54710 (17)	0.0473 (6)	
C13	0.38235 (18)	0.1214 (2)	0.6199 (2)	0.0619 (8)	
H13	0.4015	0.0758	0.6414	0.074*	
C14	0.3378 (2)	0.1713 (3)	0.6610 (2)	0.0774 (11)	
H14	0.3269	0.1583	0.7098	0.093*	
C15	0.3100 (2)	0.2393 (2)	0.6300 (3)	0.0756 (11)	
H15	0.2814	0.2727	0.6581	0.091*	
C16	0.32451 (18)	0.2576 (2)	0.5584 (2)	0.0667 (9)	
H16	0.3052	0.3034	0.5374	0.080*	
C17	0.36768 (16)	0.20877 (19)	0.51643 (19)	0.0555 (7)	
H17	0.3767	0.2218	0.4672	0.067*	
B1	0.24097 (16)	0.00116 (15)	0.37591 (15)	0.0332 (5)	
C18	0.16773 (14)	0.05003 (14)	0.40683 (13)	0.0363 (5)	
C19	0.17970 (15)	0.10436 (15)	0.46496 (14)	0.0403 (5)	
H19	0.2300	0.1110	0.4869	0.048*	
C20	0.12048 (18)	0.14858 (16)	0.49124 (15)	0.0470 (6)	
H20	0.1315	0.1841	0.5297	0.056*	
C21	0.04522 (18)	0.14003 (18)	0.46044 (17)	0.0539 (7)	
H21	0.0050	0.1691	0.4783	0.065*	
C22	0.03012 (17)	0.0878 (2)	0.4026 (2)	0.0592 (8)	
H22	−0.0205	0.0816	0.3813	0.071*	
C23	0.09034 (16)	0.04449 (18)	0.37650 (16)	0.0493 (6)	
H23	0.0789	0.0102	0.3371	0.059*	
C24	0.20756 (14)	−0.06016 (15)	0.31129 (13)	0.0364 (5)	
C25	0.18838 (16)	−0.03653 (16)	0.23707 (14)	0.0433 (6)	
H25	0.1983	0.0143	0.2240	0.052*	
C26	0.15512 (17)	−0.08579 (18)	0.18195 (15)	0.0489 (6)	
H26	0.1425	−0.0674	0.1335	0.059*	
C27	0.14083 (16)	−0.16161 (18)	0.19889 (16)	0.0489 (6)	
H27	0.1189	−0.1949	0.1622	0.059*	
C28	0.15954 (18)	−0.18736 (18)	0.27100 (17)	0.0536 (7)	
H28	0.1504	−0.2385	0.2833	0.064*	
C29	0.19205 (16)	−0.13735 (16)	0.32564 (15)	0.0451 (6)	
H29	0.2040	−0.1563	0.3740	0.054*	
C30	0.28889 (14)	−0.04720 (14)	0.44488 (13)	0.0357 (5)	
C31	0.35062 (17)	−0.09641 (17)	0.43046 (16)	0.0483 (6)	
H31	0.3626	−0.1028	0.3808	0.058*	
C32	0.39500 (18)	−0.13630 (18)	0.48623 (18)	0.0546 (7)	
H32	0.4359	−0.1676	0.4735	0.065*	
C33	0.37807 (19)	−0.12928 (18)	0.56041 (17)	0.0555 (7)	
H33	0.4077	−0.1550	0.5983	0.067*	
C34	0.31687 (18)	−0.08373 (19)	0.57725 (16)	0.0539 (7)	
H34	0.3042	−0.0793	0.6270	0.065*	
C35	0.27340 (16)	−0.04405 (16)	0.52109 (15)	0.0441 (6)	
H35	0.2320	−0.0139	0.5346	0.053*	
C36	0.30103 (14)	0.06146 (14)	0.33706 (13)	0.0339 (5)	
C37	0.36449 (15)	0.03477 (16)	0.29928 (16)	0.0474 (6)	
H37	0.3713	−0.0181	0.2946	0.057*	
C38	0.41771 (16)	0.08365 (18)	0.26854 (17)	0.0533 (7)	
H38	0.4597	0.0630	0.2453	0.064*	
C39	0.40879 (18)	0.16192 (18)	0.27215 (16)	0.0524 (7)	
H39	0.4450	0.1947	0.2526	0.063*	
C40	0.34551 (19)	0.19114 (17)	0.30517 (17)	0.0551 (7)	
H40	0.3375	0.2441	0.3066	0.066*	
C41	0.29310 (16)	0.14124 (15)	0.33662 (15)	0.0445 (6)	
H41	0.2506	0.1625	0.3585	0.053*	
B2	0.23573 (16)	0.49378 (16)	0.26613 (16)	0.0368 (6)	
C42	0.19430 (14)	0.54596 (14)	0.19683 (13)	0.0363 (5)	
C43	0.13153 (15)	0.59506 (15)	0.20904 (15)	0.0420 (5)	
H43	0.1145	0.5982	0.2573	0.050*	
C44	0.09382 (17)	0.63904 (16)	0.15267 (17)	0.0500 (6)	
H44	0.0522	0.6704	0.1635	0.060*	
C45	0.11769 (17)	0.63649 (16)	0.08052 (16)	0.0496 (7)	
H45	0.0919	0.6651	0.0422	0.060*	
C46	0.18023 (17)	0.59087 (16)	0.06629 (15)	0.0470 (6)	
H46	0.1977	0.5894	0.0181	0.056*	
C47	0.21761 (15)	0.54683 (15)	0.12338 (14)	0.0406 (5)	
H47	0.2599	0.5167	0.1121	0.049*	
C48	0.30820 (15)	0.44317 (15)	0.23630 (13)	0.0384 (5)	
C49	0.38646 (17)	0.4554 (2)	0.25770 (18)	0.0560 (7)	
H49	0.4001	0.4951	0.2912	0.067*	
C50	0.44541 (19)	0.4100 (2)	0.2306 (2)	0.0694 (9)	
H50	0.4972	0.4194	0.2469	0.083*	
C51	0.4276 (2)	0.3521 (2)	0.1805 (2)	0.0632 (9)	
H51	0.4670	0.3229	0.1615	0.076*	
C52	0.35127 (19)	0.33743 (18)	0.15840 (17)	0.0535 (7)	
H52	0.3384	0.2976	0.1248	0.064*	
C53	0.29295 (18)	0.38214 (17)	0.18621 (16)	0.0480 (6)	
H53	0.2414	0.3710	0.1708	0.058*	
C54	0.17330 (15)	0.43080 (15)	0.29723 (13)	0.0380 (5)	
C55	0.20049 (18)	0.37067 (15)	0.34500 (16)	0.0492 (6)	
H55	0.2537	0.3675	0.3584	0.059*	
C56	0.1518 (2)	0.31584 (17)	0.37310 (17)	0.0591 (8)	
H56	0.1724	0.2775	0.4052	0.071*	
C57	0.0725 (2)	0.31826 (18)	0.35339 (19)	0.0629 (9)	
H57	0.0394	0.2817	0.3720	0.076*	
C58	0.04336 (19)	0.37572 (19)	0.30576 (19)	0.0595 (7)	
H58	−0.0098	0.3778	0.2918	0.071*	
C59	0.09315 (16)	0.43037 (16)	0.27868 (15)	0.0458 (6)	
H59	0.0720	0.4685	0.2466	0.055*	
C60	0.26902 (14)	0.55380 (14)	0.33323 (14)	0.0378 (5)	
C61	0.27814 (17)	0.53473 (18)	0.40924 (15)	0.0490 (6)	
H61	0.2628	0.4860	0.4241	0.059*	
C62	0.30937 (18)	0.5856 (2)	0.46422 (16)	0.0573 (8)	
H62	0.3147	0.5702	0.5144	0.069*	
C63	0.33218 (16)	0.65778 (19)	0.44484 (17)	0.0528 (7)	
H63	0.3525	0.6919	0.4815	0.063*	
C64	0.3248 (2)	0.67914 (19)	0.37105 (19)	0.0615 (8)	
H64	0.3405	0.7280	0.3570	0.074*	
C65	0.2935 (2)	0.62760 (18)	0.31646 (17)	0.0583 (8)	
H65	0.2890	0.6436	0.2665	0.070*	

### Atomic displacement parameters (Å^2^)

**Table:**

	*U*^11^	*U*^22^	*U*^33^	*U*^12^	*U*^13^	*U*^23^
N1	0.086 (2)	0.0510 (16)	0.096 (2)	0.0063 (15)	0.0136 (17)	0.0080 (15)
N2	0.0662 (17)	0.0679 (18)	0.0758 (19)	−0.0170 (14)	0.0009 (14)	−0.0040 (15)
N3	0.0723 (18)	0.0514 (16)	0.107 (3)	−0.0109 (14)	0.0335 (18)	0.0011 (17)
N4	0.0407 (10)	0.0412 (11)	0.0462 (11)	0.0073 (9)	0.0008 (9)	−0.0046 (9)
C1	0.0577 (17)	0.0404 (15)	0.085 (2)	−0.0120 (13)	0.0093 (16)	0.0009 (15)
C2	0.143 (5)	0.083 (3)	0.112 (4)	0.016 (3)	0.002 (3)	0.039 (3)
C3	0.116 (4)	0.069 (3)	0.176 (6)	0.024 (3)	0.023 (4)	−0.019 (3)
C4	0.088 (3)	0.094 (3)	0.111 (3)	−0.015 (2)	−0.032 (3)	−0.018 (3)
C5	0.091 (3)	0.138 (4)	0.075 (3)	−0.028 (3)	0.016 (2)	0.000 (3)
C6	0.0477 (17)	0.071 (2)	0.127 (3)	−0.0041 (16)	0.011 (2)	−0.012 (2)
C7	0.0593 (19)	0.071 (2)	0.104 (3)	−0.0047 (17)	0.037 (2)	−0.006 (2)
C8	0.0512 (15)	0.0540 (17)	0.0627 (17)	0.0045 (13)	0.0123 (13)	0.0046 (14)
C9	0.0619 (17)	0.068 (2)	0.0491 (15)	0.0162 (15)	−0.0070 (13)	−0.0007 (14)
C10	0.0596 (17)	0.0620 (19)	0.0667 (19)	0.0179 (15)	0.0029 (15)	−0.0204 (16)
C11	0.0408 (13)	0.0498 (16)	0.0616 (16)	−0.0027 (12)	0.0006 (12)	−0.0148 (13)
C12	0.0326 (11)	0.0491 (15)	0.0604 (16)	−0.0037 (10)	0.0038 (11)	−0.0137 (12)
C13	0.0560 (17)	0.0615 (19)	0.0703 (19)	−0.0096 (14)	0.0189 (15)	−0.0011 (16)
C14	0.064 (2)	0.093 (3)	0.080 (2)	−0.019 (2)	0.0344 (19)	−0.015 (2)
C15	0.0500 (18)	0.076 (2)	0.104 (3)	−0.0071 (17)	0.0281 (19)	−0.036 (2)
C16	0.0444 (15)	0.066 (2)	0.089 (2)	0.0067 (14)	0.0058 (15)	−0.0163 (18)
C17	0.0406 (13)	0.0636 (18)	0.0615 (17)	0.0059 (13)	−0.0014 (12)	−0.0089 (14)
B1	0.0357 (12)	0.0337 (13)	0.0301 (11)	−0.0013 (10)	0.0027 (10)	−0.0021 (10)
C18	0.0379 (12)	0.0378 (13)	0.0340 (12)	−0.0012 (10)	0.0075 (10)	0.0015 (10)
C19	0.0414 (13)	0.0417 (13)	0.0380 (12)	−0.0012 (10)	0.0040 (10)	0.0002 (10)
C20	0.0640 (17)	0.0403 (14)	0.0381 (13)	0.0052 (12)	0.0134 (12)	0.0007 (10)
C21	0.0507 (15)	0.0534 (17)	0.0601 (18)	0.0137 (13)	0.0197 (14)	0.0031 (14)
C22	0.0375 (14)	0.069 (2)	0.0715 (19)	0.0052 (13)	0.0034 (13)	−0.0049 (17)
C23	0.0419 (14)	0.0565 (17)	0.0492 (15)	0.0004 (12)	0.0003 (12)	−0.0082 (13)
C24	0.0346 (11)	0.0398 (13)	0.0349 (12)	0.0003 (10)	0.0039 (9)	−0.0043 (10)
C25	0.0496 (14)	0.0388 (13)	0.0409 (13)	0.0016 (11)	−0.0002 (11)	−0.0012 (11)
C26	0.0523 (15)	0.0601 (18)	0.0333 (12)	0.0046 (13)	−0.0030 (11)	−0.0070 (12)
C27	0.0440 (14)	0.0548 (17)	0.0482 (15)	−0.0075 (12)	0.0047 (12)	−0.0190 (13)
C28	0.0631 (18)	0.0432 (15)	0.0551 (16)	−0.0133 (13)	0.0077 (14)	−0.0068 (13)
C29	0.0522 (15)	0.0451 (15)	0.0384 (13)	−0.0085 (12)	0.0060 (11)	−0.0001 (11)
C30	0.0368 (12)	0.0345 (12)	0.0354 (12)	−0.0060 (9)	−0.0001 (9)	−0.0006 (10)
C31	0.0530 (15)	0.0500 (16)	0.0419 (14)	0.0098 (13)	0.0029 (12)	0.0042 (12)
C32	0.0517 (15)	0.0484 (16)	0.0625 (18)	0.0083 (13)	−0.0033 (13)	0.0062 (14)
C33	0.0585 (17)	0.0534 (17)	0.0525 (17)	−0.0031 (14)	−0.0115 (13)	0.0152 (13)
C34	0.0628 (17)	0.0601 (18)	0.0381 (13)	−0.0071 (14)	−0.0001 (12)	0.0083 (13)
C35	0.0455 (14)	0.0467 (14)	0.0403 (13)	−0.0033 (11)	0.0038 (11)	0.0038 (11)
C36	0.0360 (11)	0.0370 (12)	0.0282 (10)	−0.0011 (9)	−0.0014 (9)	0.0005 (9)
C37	0.0469 (14)	0.0417 (14)	0.0552 (15)	0.0063 (11)	0.0141 (12)	0.0029 (12)
C38	0.0442 (14)	0.0621 (18)	0.0555 (15)	0.0048 (13)	0.0168 (12)	0.0110 (14)
C39	0.0563 (16)	0.0561 (18)	0.0461 (14)	−0.0115 (13)	0.0121 (12)	0.0102 (13)
C40	0.0746 (19)	0.0387 (14)	0.0538 (15)	0.0000 (13)	0.0174 (14)	0.0077 (12)
C41	0.0489 (14)	0.0408 (14)	0.0449 (13)	0.0073 (11)	0.0109 (11)	0.0038 (11)
B2	0.0418 (14)	0.0347 (14)	0.0337 (12)	0.0018 (11)	0.0025 (11)	−0.0006 (11)
C42	0.0403 (13)	0.0325 (12)	0.0362 (12)	−0.0051 (9)	0.0024 (10)	−0.0008 (9)
C43	0.0460 (13)	0.0387 (13)	0.0418 (13)	−0.0004 (11)	0.0050 (11)	0.0009 (11)
C44	0.0464 (14)	0.0409 (15)	0.0620 (17)	0.0007 (12)	−0.0021 (13)	0.0055 (13)
C45	0.0568 (16)	0.0402 (14)	0.0498 (15)	−0.0105 (12)	−0.0106 (12)	0.0123 (11)
C46	0.0628 (16)	0.0431 (14)	0.0352 (13)	−0.0133 (13)	0.0037 (12)	0.0065 (11)
C47	0.0465 (13)	0.0374 (13)	0.0382 (12)	−0.0045 (10)	0.0059 (11)	−0.0005 (10)
C48	0.0419 (13)	0.0384 (13)	0.0350 (12)	0.0005 (10)	0.0040 (10)	0.0047 (10)
C49	0.0453 (15)	0.0607 (19)	0.0626 (18)	−0.0022 (13)	0.0086 (13)	−0.0132 (15)
C50	0.0424 (15)	0.073 (2)	0.094 (3)	0.0026 (15)	0.0150 (16)	−0.009 (2)
C51	0.0595 (19)	0.061 (2)	0.071 (2)	0.0196 (15)	0.0239 (17)	0.0050 (16)
C52	0.0687 (19)	0.0455 (15)	0.0469 (15)	0.0140 (14)	0.0080 (14)	−0.0020 (12)
C53	0.0514 (14)	0.0448 (14)	0.0472 (14)	0.0068 (12)	0.0000 (12)	−0.0019 (12)
C54	0.0472 (13)	0.0335 (12)	0.0340 (12)	0.0015 (10)	0.0080 (10)	−0.0026 (9)
C55	0.0613 (16)	0.0378 (14)	0.0493 (15)	0.0052 (12)	0.0087 (13)	0.0041 (11)
C56	0.089 (2)	0.0367 (14)	0.0537 (17)	0.0035 (14)	0.0220 (16)	0.0070 (12)
C57	0.085 (2)	0.0439 (16)	0.0645 (19)	−0.0135 (15)	0.0340 (17)	−0.0001 (14)
C58	0.0527 (16)	0.0587 (18)	0.0691 (19)	−0.0081 (14)	0.0181 (14)	−0.0049 (15)
C59	0.0487 (14)	0.0417 (13)	0.0478 (14)	−0.0011 (11)	0.0099 (11)	0.0007 (11)
C60	0.0387 (12)	0.0368 (13)	0.0378 (12)	0.0032 (10)	0.0021 (10)	−0.0017 (10)
C61	0.0574 (16)	0.0493 (16)	0.0407 (14)	−0.0009 (13)	0.0074 (12)	−0.0011 (12)
C62	0.0583 (17)	0.077 (2)	0.0373 (14)	−0.0025 (16)	0.0056 (12)	−0.0106 (14)
C63	0.0436 (14)	0.0588 (18)	0.0554 (17)	0.0030 (13)	0.0000 (13)	−0.0204 (14)
C64	0.069 (2)	0.0433 (16)	0.070 (2)	−0.0066 (14)	−0.0096 (16)	−0.0057 (14)
C65	0.082 (2)	0.0446 (16)	0.0460 (15)	−0.0094 (15)	−0.0107 (15)	0.0051 (12)

### Geometric parameters (Å, º)

**Table:**

N1—C1	1.336 (5)	C27—H27	0.9300
N1—C2	1.464 (6)	C28—C29	1.390 (4)
N1—C3	1.484 (6)	C28—H28	0.9300
N2—C1	1.323 (4)	C29—H29	0.9300
N2—C5	1.468 (5)	C30—C31	1.401 (4)
N2—C4	1.472 (5)	C30—C35	1.403 (3)
N3—C1	1.337 (5)	C31—C32	1.392 (4)
N3—C6	1.452 (5)	C31—H31	0.9300
N3—H3	0.85 (5)	C32—C33	1.379 (4)
N4—C9	1.483 (4)	C32—H32	0.9300
N4—C10	1.508 (3)	C33—C34	1.367 (5)
N4—C8	1.510 (4)	C33—H33	0.9300
N4—C11	1.536 (3)	C34—C35	1.387 (4)
C2—H2A	0.9600	C34—H34	0.9300
C2—H2B	0.9600	C35—H35	0.9300
C2—H2C	0.9600	C36—C41	1.391 (4)
C3—H3A	0.9600	C36—C37	1.402 (3)
C3—H3B	0.9600	C37—C38	1.389 (4)
C3—H3C	0.9600	C37—H37	0.9300
C4—H4A	0.9600	C38—C39	1.368 (4)
C4—H4B	0.9600	C38—H38	0.9300
C4—H4C	0.9600	C39—C40	1.371 (4)
C5—H5A	0.9600	C39—H39	0.9300
C5—H5B	0.9600	C40—C41	1.396 (4)
C5—H5C	0.9600	C40—H40	0.9300
C6—C7	1.517 (6)	C41—H41	0.9300
C6—H6A	0.9700	B2—C48	1.644 (4)
C6—H6B	0.9700	B2—C42	1.650 (4)
C7—C8	1.519 (4)	B2—C54	1.655 (4)
C7—H7A	0.9700	B2—C60	1.655 (4)
C7—H7B	0.9700	C42—C47	1.396 (3)
C8—H8A	0.9700	C42—C43	1.404 (4)
C8—H8B	0.9700	C43—C44	1.384 (4)
C9—H9A	0.9600	C43—H43	0.9300
C9—H9B	0.9600	C44—C45	1.378 (4)
C9—H9C	0.9600	C44—H44	0.9300
C10—H10A	0.9600	C45—C46	1.374 (4)
C10—H10B	0.9600	C45—H45	0.9300
C10—H10C	0.9600	C46—C47	1.391 (4)
C11—C12	1.503 (4)	C46—H46	0.9300
C11—H11B	0.9700	C47—H47	0.9300
C11—H11A	0.9700	C48—C49	1.388 (4)
C12—C13	1.382 (4)	C48—C53	1.397 (4)
C12—C17	1.387 (4)	C49—C50	1.397 (4)
C13—C14	1.397 (5)	C49—H49	0.9300
C13—H13	0.9300	C50—C51	1.364 (5)
C14—C15	1.374 (6)	C50—H50	0.9300
C14—H14	0.9300	C51—C52	1.367 (5)
C15—C16	1.354 (6)	C51—H51	0.9300
C15—H15	0.9300	C52—C53	1.388 (4)
C16—C17	1.381 (4)	C52—H52	0.9300
C16—H16	0.9300	C53—H53	0.9300
C17—H17	0.9300	C54—C59	1.394 (4)
B1—C24	1.640 (3)	C54—C55	1.405 (4)
B1—C18	1.646 (4)	C55—C56	1.385 (4)
B1—C36	1.656 (3)	C55—H55	0.9300
B1—C30	1.657 (4)	C56—C57	1.384 (5)
C18—C23	1.403 (4)	C56—H56	0.9300
C18—C19	1.404 (3)	C57—C58	1.379 (5)
C19—C20	1.384 (4)	C57—H57	0.9300
C19—H19	0.9300	C58—C59	1.387 (4)
C20—C21	1.377 (5)	C58—H58	0.9300
C20—H20	0.9300	C59—H59	0.9300
C21—C22	1.382 (5)	C60—C65	1.387 (4)
C21—H21	0.9300	C60—C61	1.391 (4)
C22—C23	1.387 (4)	C61—C62	1.397 (4)
C22—H22	0.9300	C61—H61	0.9300
C23—H23	0.9300	C62—C63	1.363 (5)
C24—C29	1.393 (4)	C62—H62	0.9300
C24—C25	1.400 (4)	C63—C64	1.362 (5)
C25—C26	1.392 (4)	C63—H63	0.9300
C25—H25	0.9300	C64—C65	1.399 (4)
C26—C27	1.375 (4)	C64—H64	0.9300
C26—H26	0.9300	C65—H65	0.9300
C27—C28	1.375 (4)		
			
C1—N1—C2	122.8 (4)	C27—C26—C25	120.2 (3)
C1—N1—C3	121.3 (4)	C27—C26—H26	119.9
C2—N1—C3	115.2 (4)	C25—C26—H26	119.9
C1—N2—C5	122.1 (3)	C28—C27—C26	118.8 (3)
C1—N2—C4	123.1 (3)	C28—C27—H27	120.6
C5—N2—C4	114.7 (4)	C26—C27—H27	120.6
C1—N3—C6	127.1 (4)	C27—C28—C29	120.4 (3)
C1—N3—H3	113 (3)	C27—C28—H28	119.8
C6—N3—H3	120 (3)	C29—C28—H28	119.8
C9—N4—C10	109.1 (2)	C28—C29—C24	123.0 (3)
C9—N4—C8	112.2 (2)	C28—C29—H29	118.5
C10—N4—C8	110.0 (2)	C24—C29—H29	118.5
C9—N4—C11	110.5 (2)	C31—C30—C35	113.6 (2)
C10—N4—C11	106.3 (2)	C31—C30—B1	121.0 (2)
C8—N4—C11	108.7 (2)	C35—C30—B1	125.3 (2)
N2—C1—N1	121.4 (3)	C32—C31—C30	123.8 (3)
N2—C1—N3	120.9 (3)	C32—C31—H31	118.1
N1—C1—N3	117.7 (4)	C30—C31—H31	118.1
N1—C2—H2A	109.5	C33—C32—C31	119.8 (3)
N1—C2—H2B	109.5	C33—C32—H32	120.1
H2A—C2—H2B	109.5	C31—C32—H32	120.1
N1—C2—H2C	109.5	C34—C33—C32	118.8 (3)
H2A—C2—H2C	109.5	C34—C33—H33	120.6
H2B—C2—H2C	109.5	C32—C33—H33	120.6
N1—C3—H3A	109.5	C33—C34—C35	120.8 (3)
N1—C3—H3B	109.5	C33—C34—H34	119.6
H3A—C3—H3B	109.5	C35—C34—H34	119.6
N1—C3—H3C	109.5	C34—C35—C30	123.2 (3)
H3A—C3—H3C	109.5	C34—C35—H35	118.4
H3B—C3—H3C	109.5	C30—C35—H35	118.4
N2—C4—H4A	109.5	C41—C36—C37	113.9 (2)
N2—C4—H4B	109.5	C41—C36—B1	124.5 (2)
H4A—C4—H4B	109.5	C37—C36—B1	121.5 (2)
N2—C4—H4C	109.5	C38—C37—C36	123.1 (3)
H4A—C4—H4C	109.5	C38—C37—H37	118.5
H4B—C4—H4C	109.5	C36—C37—H37	118.5
N2—C5—H5A	109.5	C39—C38—C37	120.5 (3)
N2—C5—H5B	109.5	C39—C38—H38	119.7
H5A—C5—H5B	109.5	C37—C38—H38	119.7
N2—C5—H5C	109.5	C38—C39—C40	118.8 (3)
H5A—C5—H5C	109.5	C38—C39—H39	120.6
H5B—C5—H5C	109.5	C40—C39—H39	120.6
N3—C6—C7	112.5 (4)	C39—C40—C41	119.9 (3)
N3—C6—H6A	109.1	C39—C40—H40	120.0
C7—C6—H6A	109.1	C41—C40—H40	120.0
N3—C6—H6B	109.1	C36—C41—C40	123.6 (2)
C7—C6—H6B	109.1	C36—C41—H41	118.2
H6A—C6—H6B	107.8	C40—C41—H41	118.2
C6—C7—C8	114.2 (3)	C48—B2—C42	110.3 (2)
C6—C7—H7A	108.7	C48—B2—C54	106.3 (2)
C8—C7—H7A	108.7	C42—B2—C54	110.9 (2)
C6—C7—H7B	108.7	C48—B2—C60	109.8 (2)
C8—C7—H7B	108.7	C42—B2—C60	107.6 (2)
H7A—C7—H7B	107.6	C54—B2—C60	111.9 (2)
N4—C8—C7	117.1 (3)	C47—C42—C43	114.5 (2)
N4—C8—H8A	108.0	C47—C42—B2	124.7 (2)
C7—C8—H8A	108.0	C43—C42—B2	120.8 (2)
N4—C8—H8B	108.0	C44—C43—C42	123.1 (2)
C7—C8—H8B	108.0	C44—C43—H43	118.4
H8A—C8—H8B	107.3	C42—C43—H43	118.4
N4—C9—H9A	109.5	C45—C44—C43	120.2 (3)
N4—C9—H9B	109.5	C45—C44—H44	119.9
H9A—C9—H9B	109.5	C43—C44—H44	119.9
N4—C9—H9C	109.5	C46—C45—C44	118.8 (3)
H9A—C9—H9C	109.5	C46—C45—H45	120.6
H9B—C9—H9C	109.5	C44—C45—H45	120.6
N4—C10—H10A	109.5	C45—C46—C47	120.5 (3)
N4—C10—H10B	109.5	C45—C46—H46	119.7
H10A—C10—H10B	109.5	C47—C46—H46	119.7
N4—C10—H10C	109.5	C46—C47—C42	122.8 (3)
H10A—C10—H10C	109.5	C46—C47—H47	118.6
H10B—C10—H10C	109.5	C42—C47—H47	118.6
C12—C11—N4	115.6 (2)	C49—C48—C53	115.1 (3)
C12—C11—H11B	108.4	C49—C48—B2	124.9 (2)
N4—C11—H11B	108.4	C53—C48—B2	120.0 (2)
C12—C11—H11A	108.4	C48—C49—C50	122.2 (3)
N4—C11—H11A	108.4	C48—C49—H49	118.9
H11B—C11—H11A	107.5	C50—C49—H49	118.9
C13—C12—C17	118.9 (3)	C51—C50—C49	120.5 (3)
C13—C12—C11	120.5 (3)	C51—C50—H50	119.8
C17—C12—C11	120.2 (3)	C49—C50—H50	119.8
C12—C13—C14	119.5 (4)	C50—C51—C52	119.4 (3)
C12—C13—H13	120.2	C50—C51—H51	120.3
C14—C13—H13	120.2	C52—C51—H51	120.3
C15—C14—C13	120.6 (4)	C51—C52—C53	119.8 (3)
C15—C14—H14	119.7	C51—C52—H52	120.1
C13—C14—H14	119.7	C53—C52—H52	120.1
C16—C15—C14	119.8 (3)	C52—C53—C48	123.0 (3)
C16—C15—H15	120.1	C52—C53—H53	118.5
C14—C15—H15	120.1	C48—C53—H53	118.5
C15—C16—C17	120.6 (4)	C59—C54—C55	114.8 (2)
C15—C16—H16	119.7	C59—C54—B2	125.4 (2)
C17—C16—H16	119.7	C55—C54—B2	119.8 (2)
C16—C17—C12	120.6 (3)	C56—C55—C54	123.1 (3)
C16—C17—H17	119.7	C56—C55—H55	118.5
C12—C17—H17	119.7	C54—C55—H55	118.5
C24—B1—C18	109.3 (2)	C57—C56—C55	119.9 (3)
C24—B1—C36	108.29 (19)	C57—C56—H56	120.1
C18—B1—C36	109.28 (19)	C55—C56—H56	120.1
C24—B1—C30	108.77 (19)	C58—C57—C56	119.0 (3)
C18—B1—C30	111.36 (19)	C58—C57—H57	120.5
C36—B1—C30	109.75 (19)	C56—C57—H57	120.5
C23—C18—C19	114.4 (2)	C57—C58—C59	120.1 (3)
C23—C18—B1	124.3 (2)	C57—C58—H58	119.9
C19—C18—B1	121.2 (2)	C59—C58—H58	119.9
C20—C19—C18	123.3 (3)	C58—C59—C54	123.1 (3)
C20—C19—H19	118.3	C58—C59—H59	118.5
C18—C19—H19	118.3	C54—C59—H59	118.5
C21—C20—C19	120.0 (3)	C65—C60—C61	114.5 (2)
C21—C20—H20	120.0	C65—C60—B2	121.3 (2)
C19—C20—H20	120.0	C61—C60—B2	124.2 (2)
C20—C21—C22	119.2 (3)	C60—C61—C62	122.8 (3)
C20—C21—H21	120.4	C60—C61—H61	118.6
C22—C21—H21	120.4	C62—C61—H61	118.6
C21—C22—C23	120.1 (3)	C63—C62—C61	120.4 (3)
C21—C22—H22	120.0	C63—C62—H62	119.8
C23—C22—H22	120.0	C61—C62—H62	119.8
C22—C23—C18	123.0 (3)	C64—C63—C62	119.0 (3)
C22—C23—H23	118.5	C64—C63—H63	120.5
C18—C23—H23	118.5	C62—C63—H63	120.5
C29—C24—C25	114.8 (2)	C63—C64—C65	120.1 (3)
C29—C24—B1	123.8 (2)	C63—C64—H64	120.0
C25—C24—B1	121.3 (2)	C65—C64—H64	120.0
C26—C25—C24	122.8 (3)	C60—C65—C64	123.2 (3)
C26—C25—H25	118.6	C60—C65—H65	118.4
C24—C25—H25	118.6	C64—C65—H65	118.4
			
C5—N2—C1—N1	31.1 (5)	C24—B1—C36—C41	123.8 (3)
C4—N2—C1—N1	−146.3 (4)	C18—B1—C36—C41	4.7 (3)
C5—N2—C1—N3	−147.9 (4)	C30—B1—C36—C41	−117.7 (3)
C4—N2—C1—N3	34.8 (5)	C24—B1—C36—C37	−54.5 (3)
C2—N1—C1—N2	−154.0 (4)	C18—B1—C36—C37	−173.5 (2)
C3—N1—C1—N2	36.2 (5)	C30—B1—C36—C37	64.1 (3)
C2—N1—C1—N3	25.0 (5)	C41—C36—C37—C38	4.1 (4)
C3—N1—C1—N3	−144.8 (4)	B1—C36—C37—C38	−177.5 (2)
C6—N3—C1—N2	32.7 (5)	C36—C37—C38—C39	−1.9 (5)
C6—N3—C1—N1	−146.3 (4)	C37—C38—C39—C40	−1.4 (5)
C1—N3—C6—C7	108.1 (5)	C38—C39—C40—C41	2.2 (5)
N3—C6—C7—C8	−63.3 (5)	C37—C36—C41—C40	−3.3 (4)
C9—N4—C8—C7	55.6 (4)	B1—C36—C41—C40	178.4 (3)
C10—N4—C8—C7	−66.0 (3)	C39—C40—C41—C36	0.3 (5)
C11—N4—C8—C7	178.0 (3)	C48—B2—C42—C47	0.0 (3)
C6—C7—C8—N4	−101.2 (4)	C54—B2—C42—C47	−117.5 (3)
C9—N4—C11—C12	62.6 (3)	C60—B2—C42—C47	119.8 (2)
C10—N4—C11—C12	−179.2 (3)	C48—B2—C42—C43	−179.0 (2)
C8—N4—C11—C12	−60.9 (3)	C54—B2—C42—C43	63.4 (3)
N4—C11—C12—C13	−96.1 (3)	C60—B2—C42—C43	−59.2 (3)
N4—C11—C12—C17	91.1 (3)	C47—C42—C43—C44	2.0 (4)
C17—C12—C13—C14	−0.5 (4)	B2—C42—C43—C44	−178.8 (2)
C11—C12—C13—C14	−173.4 (3)	C42—C43—C44—C45	−0.5 (4)
C12—C13—C14—C15	−0.7 (5)	C43—C44—C45—C46	−1.3 (4)
C13—C14—C15—C16	1.3 (5)	C44—C45—C46—C47	1.4 (4)
C14—C15—C16—C17	−0.6 (5)	C45—C46—C47—C42	0.3 (4)
C15—C16—C17—C12	−0.7 (5)	C43—C42—C47—C46	−1.9 (4)
C13—C12—C17—C16	1.2 (4)	B2—C42—C47—C46	179.0 (2)
C11—C12—C17—C16	174.1 (3)	C42—B2—C48—C49	111.4 (3)
C24—B1—C18—C23	−5.8 (3)	C54—B2—C48—C49	−128.3 (3)
C36—B1—C18—C23	112.6 (3)	C60—B2—C48—C49	−7.1 (4)
C30—B1—C18—C23	−126.0 (3)	C42—B2—C48—C53	−69.4 (3)
C24—B1—C18—C19	177.3 (2)	C54—B2—C48—C53	51.0 (3)
C36—B1—C18—C19	−64.4 (3)	C60—B2—C48—C53	172.2 (2)
C30—B1—C18—C19	57.0 (3)	C53—C48—C49—C50	0.3 (4)
C23—C18—C19—C20	0.6 (4)	B2—C48—C49—C50	179.6 (3)
B1—C18—C19—C20	177.9 (2)	C48—C49—C50—C51	1.1 (6)
C18—C19—C20—C21	0.4 (4)	C49—C50—C51—C52	−1.7 (6)
C19—C20—C21—C22	−0.9 (4)	C50—C51—C52—C53	1.0 (5)
C20—C21—C22—C23	0.2 (5)	C51—C52—C53—C48	0.5 (5)
C21—C22—C23—C18	0.9 (5)	C49—C48—C53—C52	−1.1 (4)
C19—C18—C23—C22	−1.3 (4)	B2—C48—C53—C52	179.5 (3)
B1—C18—C23—C22	−178.5 (3)	C48—B2—C54—C59	−131.4 (2)
C18—B1—C24—C29	−96.4 (3)	C42—B2—C54—C59	−11.4 (3)
C36—B1—C24—C29	144.6 (2)	C60—B2—C54—C59	108.7 (3)
C30—B1—C24—C29	25.4 (3)	C48—B2—C54—C55	47.0 (3)
C18—B1—C24—C25	80.0 (3)	C42—B2—C54—C55	167.0 (2)
C36—B1—C24—C25	−39.0 (3)	C60—B2—C54—C55	−72.8 (3)
C30—B1—C24—C25	−158.2 (2)	C59—C54—C55—C56	−1.3 (4)
C29—C24—C25—C26	1.1 (4)	B2—C54—C55—C56	−179.9 (3)
B1—C24—C25—C26	−175.7 (2)	C54—C55—C56—C57	0.9 (4)
C24—C25—C26—C27	−1.1 (4)	C55—C56—C57—C58	0.0 (5)
C25—C26—C27—C28	0.4 (4)	C56—C57—C58—C59	−0.4 (5)
C26—C27—C28—C29	0.2 (4)	C57—C58—C59—C54	0.0 (5)
C27—C28—C29—C24	−0.2 (5)	C55—C54—C59—C58	0.8 (4)
C25—C24—C29—C28	−0.4 (4)	B2—C54—C59—C58	179.3 (3)
B1—C24—C29—C28	176.2 (3)	C48—B2—C60—C65	89.8 (3)
C24—B1—C30—C31	55.6 (3)	C42—B2—C60—C65	−30.3 (3)
C18—B1—C30—C31	176.2 (2)	C54—B2—C60—C65	−152.4 (3)
C36—B1—C30—C31	−62.7 (3)	C48—B2—C60—C61	−87.3 (3)
C24—B1—C30—C35	−124.4 (3)	C42—B2—C60—C61	152.6 (2)
C18—B1—C30—C35	−3.8 (3)	C54—B2—C60—C61	30.5 (3)
C36—B1—C30—C35	117.3 (3)	C65—C60—C61—C62	0.0 (4)
C35—C30—C31—C32	−2.5 (4)	B2—C60—C61—C62	177.3 (3)
B1—C30—C31—C32	177.5 (3)	C60—C61—C62—C63	0.4 (5)
C30—C31—C32—C33	1.1 (5)	C61—C62—C63—C64	−0.7 (5)
C31—C32—C33—C34	0.9 (5)	C62—C63—C64—C65	0.5 (5)
C32—C33—C34—C35	−1.2 (5)	C61—C60—C65—C64	−0.1 (5)
C33—C34—C35—C30	−0.4 (5)	B2—C60—C65—C64	−177.5 (3)
C31—C30—C35—C34	2.2 (4)	C63—C64—C65—C60	−0.1 (5)
B1—C30—C35—C34	−177.8 (3)		

### Hydrogen-bond geometry (Å, º)

Cg1–Cg5 are the centroids of the C36–C41, C30–C35, C24–C29, C42–C47 and C60-C65 rings, respectively.

**Table:**

*D*—H···*A*	*D*—H	H···*A*	*D*···*A*	*D*—H···*A*
C11—H11*B*···*Cg*1	0.97	2.73	3.699 (2)	174
C11—H11*A*···*Cg*2	0.97	2.66	3.509 (2)	145
C14—H14···*Cg*3^i^	0.93	2.92	3.531 (2)	124
C9—H9*A*···*Cg*4^ii^	0.97	2.91	3.569 (2)	126
C7—H7*A*···*Cg*5^iii^	0.96	2.82	3.696 (2)	133

Symmetry codes: (i) *x*, −*y*, *z*+1/2; (ii) *x*+1/2, −*y*+1/2, *z*+1/2; (iii) *x*+1/2, *y*−1/2, *z*.
